# Stimulants in the Army

**Published:** 1863-10

**Authors:** James Bryan

**Affiliations:** Surg. U. S. V., Department of the Tennessee; Vicksburg


### STIMULANTS IN THE ARMY.

[To the Editor of the AMERICAN MEDICAL TIMES.]

VICKSBURG, August 1st, 1863.

SIR:—In your number of July 11th, I was gratified to read a discussion on the subject of the use of alcoholic stimulants, in the treatment of pulmonary diseases. Also an editorial by yourself, headed "An Indolent Profession."

The subject first mentioned, as you justly remark in another place, is of great importance in its relation to the habits of the people. Unfortunately the discussion of this and similar questions is sure to be influenced by the habits and preconceived notions of the disputants. Dr. Davis, of Chicago, whose advocacy of total abstinence is well known to the public and to the profession, shows by statistics that tuberculosis is very common among those who have used alcoholic drinks as a beverage or a medicine; while not one of Dr. Flint's cases, sixty in number, acquired a craving for stimulants. Dr. Parker cautions us strongly against the practice of whiskey-drinking, and Drs. Blakeman and Post give cases in which the habit of intemperance was formed from the prescription of a physician. These habits were followed by an ignominious death and a dishonored grave. I suppose that any physician in general practice, who did not use these stimulants himself as a beverage, would give the same testimony. I am sorry to hear you say that habits of intemperance are increasing rapidly amongst the people. During the last two years I have lived pretty much amongst the camps, and know but little of the condition of society. My life in the army has, however, given me an opportunity of making observations on the use, medical and general, of these stimulants among soldiers.

The appetite for stimulants in this department is certainly very strong, and is due, perhaps, first, to the effects of the intense and prolonged heat, producing great prostration ; second to want of variety in food ; and, third, to the active agency of the malaria so common in this district. The intense heat calls for stimulants ; the uniform rations, generally very salt, call for drinks, as does the free and general perspiration induced by the heat. The continued cause of poison by malaria, producing diarrhœas, gastralgias, mental, moral, and physical depression, seems to call imperatively for alcoholic stimulants. And what are we to do ? The remedy is often at hand, and the relief, though temporary, is immediate. With the idea of a malarial influence entering the system through the digestive organs, I early adopted the plan of using freely vegetable acids particularly citric, as a beverage. I found that the water which I drank was corrected by it, the gastralgia and sense of prostration relieved, while the frequent recurring diarrhœas were often prevented or cured. In charge of a large hospital at Grand Gulf, I recommended this practice to my medical staff, and they adopted it both individually and in their practice.—Without it the water we drank would produce diarrhœa in less than an hour. Surgeon Robarts, of the hospital-boat belonging to the marine fleet, has adopted the practice, and has his officers and patients freely supplied with lemonade made of citric acid, sugar and water. Several surgeons in the field have informed me that they do the same thing in their regimental hospitals, and all with good effect. It is very common amongst the officers of the army, especially in the Southern Department, to carry with them a supply of what they call "good old Bourbon," which they imbibe statedly in quantities proportioned to the sensibilities of the stomach, as a prophylactic. They often, in addition to this carry with them a quantity of morphia or pulverized opium, which is to be used as a *dernier resort.* Now, I have found that whenever an officer with these habits contracts diarrhœa or fever, it is ten times more difficult to stop it and cure him than it is to cure the same disease in one that does not use that prophylactic. The truth is, the evil in these prophylactics, to wit, the subsequent prostration of the stomach and other digestive organs, quite overbalances the temporary good obtained. To secure good health, and prevent the accession of disease, good and continuous digestion is necessary. This is sure to be interrupted by the aforesaid prophylactics. In my opinion the only way to prevent such prostration following the use of these stimulants is, to take them in small quantities, and always accompanied with some article of nutrition, such as milk, soup, bread, or crackers, and cheese, or something of the kind. In that case the stimulant acts as a digestive, and the prostration which would have followed its use is prevented by the absorption of the nutritious matter. But I have strong doubt of the propriety of using alcoholic stimulants in any case as prophylactics. The remedy, in its effects, too often becomes worse than the disease.

*Alcoholic Stimulants as Medicines.*—I claim the right to use any article I can find in the animal, vegetable or mineral kingdom, as a medicinal agent; but I would use these remedies with great caution, and always with the same restrictions that I administer narcotics and cerebral stimulants generally. I have accomplished great good by the judicious use of alcoholic stimulants in the hospital practice of the army. Convalescence from typhoid fever is often best treated by the use of these stimulants, and debility from various other causes may be removed according to the principles above suggested. The fact should always be kept clearly in mind that a stimulant is not tonic, but exhausting, unless followed by an improvement in the digestive organs. The physician at the same time is bound to respect the organization of his patient, and not (if possible) in curing him of one disease, lay the foundation of another and worse one.

Finally, I am surprised to hear you speak of an "indolent profession," and complain that medical men do not contribute to the literature of their profession. I supposed that your columns were continually supplied with original articles, both from the army and from private practice. I seldom see a medical journal, and cannot judge for myself. I know there are large numbers of well educated literary men in the army who no doubt, daily record what they see and practice in camp and hospital. These things will be published "when this cruel war is over." The Surgeon-General has taken measures, which will, no doubt, be effective, to secure an official record of the Medical Department of the Army in good form and due time. Gentlemen are, probably, occupied with these compositions, and contribute less to the journals than they otherwise would. In my opinion, a good record of military surgery is all that has been wanted to bring the medical literature of this country up to the European standard. This is now being done, and henceforth our medical, like our monetary resources, will not depend upon foreign speculators.

Yours, etc.,

JAMES BRYAN, *Surg. U. S. V.*

*Department of the Tennessee.*

